# ﻿Mitochondrial DNA 16S region and voucher specimen collection of Japanese aquatic Coleoptera and Hemiptera for environmental DNA metabarcoding analyses

**DOI:** 10.3897/zookeys.1253.146226

**Published:** 2025-09-23

**Authors:** Naoyuki Nakahama, Kei Hirasawa, Masaya Kato, Kohei Watanabe, Seikan Kurata, Masakazu Hayashi

**Affiliations:** 1 Institute of Natural and Environmental Sciences, University of Hyogo, Sanda City, Hyogo, 669–1546, Japan University of Hyogo Sanda City Japan; 2 Museum of Nature and Human Activities, Hyogo, 6 Yayoigaoka, Sanda Hyogo, 669–1546, Japan Museum of Nature and Human Activities Sanda Hyogo Japan; 3 Aquamarine Inawashiro Kingfishers Aquarium, Inawashiro Town, Fukushima, 969–3283, Japan Aquamarine Inawashiro Kingfishers Aquarium Fukushima Japan; 4 Graduate School of Agriculture, Osaka Metropolitan University, Sakai City, Osaka, 599–8531, Japan Osaka Metropolitan University Osaka Japan; 5 Ishikawa Insect Museum, Hakusan City, Ishikawa, 920–2113, Japan Ishikawa Insect Museum Ishikawa Japan; 6 Tomakomai Experimental Forest, Field Science Center for Northern Biosphere, Hokkaido University, Tomakomai City, Hokkaido, 053–0035, Japan Hokkaido University Hokkaido Japan; 7 Hoshizaki Green Foundation, Sono, Izumo, 691–0076, Japan Hoshizaki Green Foundation Izumo Japan

**Keywords:** Aquatic insects, distribution survey, DNA barcoding, eDNA, high-throughput sequencer, lentic water, mitochondrial DNA

## Abstract

Aquatic coleopteran and hemipteran insects primarily inhabit lentic waters, many of which are at risk of extinction due to development, agriculture, and invasive alien species. Environmental DNA (eDNA) analysis has recently emerged as a powerful tool for conducting comprehensive distribution surveys. The cytochrome *c* oxidase subunit I (COI) universal primers are conventionally used for DNA barcoding but they often result in non-specific amplification and frequent amplication failures. Primers in the mitochondrial DNA (mtDNA) 16S rRNA region that alleviate these issues have been developed and are considered helpful for eDNA analysis. It is necessary to accumulate reference sequences of the mtDNA 16S rRNA region in aquatic coleopteran and hemipteran insects. However, molecular identification at the genus or species level remains challenging, as only a few of these insect groups in Japan have registered reference DNA sequences for both the mtDNACOI and 16S rRNA. Therefore, we constructed a comprehensive dataset of the mtDNA 16S rRNA region for these insects distributed in Japan. As a result of this study, we were able to obtain partial sequences of the mtDNA 16S rRNA region from 140 coleopteran taxa (35.5% of Japanese aquatic species or subspecies) and 58 hemipteran taxa (45.3% of Japanese aquatic species or subspecies). These voucher specimens were deposited in four research institutions. The DNA sequence datasets are expected to significantly contribute as an essential database for eDNA analysis and other DNA metabarcoding studies.

## ﻿Introduction

Aquatic insects, of which approximately 100,000 species have been described, include many taxonomic groups such as Coleoptera, Diptera, Ephemeroptera, Hemiptera, Megaloptera, Neuroptera, Odonata, Plecoptera, and Trichoptera (Kawai and Tanida 2018). They play vital roles in maintaining ecosystems (Dijkstra et al. 2014; Macadam and Stockan 2015; Raitif et al. 2019). For example, they drive nutrient cycles in terrestrial and aquatic environments and serve as predators of vectors of infectious diseases (Hupfer et al. 2019; Dambach 2020). Therefore, the conservation of aquatic insect diversity is a critical global issue (Macadam and Stockan 2015). However, wetland ecosystems are declining worldwide, and many aquatic insects are threatened with extinction (Collier et al. 2016; IUCN 2024). Of the aquatic invertebrates, 34% of all taxa were ranked as critically endangered, endangered, or near threatened (Collen et al. 2012). As many of these threats stem from human activities, such as land development, invasive alien species, pesticides, and climate change (Collier et al. 2016; IUCN 2024), aquatic insect diversity will continue to decline in the future unless conservation efforts are implemented.

Here, we focused on aquatic Coleoptera and Hemiptera, which spend almost their entire life cycle in or on the water (Kawai and Tanida 2018; Nakajima et al. 2020). Many of these are insectivores and provide various ecosystem services, such as controlling outbreaks of blood-sucking pests (Saha et al. 2010; Nakajima et al. 2020; Vanslembrouck et al. 2024). In Japan, as many aquatic insects, especially Coleoptera and Hemiptera, are critically threatened, it is imperative to monitor their distribution and conservation efforts (Ministry of the Environment, Government of Japan 2014; Hayashi et al. 2020; Nakajima et al. 2020). In total, 30.4% of these aquatic coleopteran insects and 24.6% of aquatic hemipteran insects are listed in various ranks on Japan’s Ministry of Environment’s Red List, 2019 (Hayashi et al. 2020). The risk and urgency primarily arise because many of these groups inhabit lentic waters. More than 60% of both aquatic coleopteran and hemipteran insect species reside in lentic waters such as rice paddies, ponds, and puddles (Hayashi et al. 2020; Nakajima et al. 2020). The abandonment or intensification of paddies and ponds, along with the presence of alien species and pesticides, leads to population decline (Gherardi and Acquistapace 2007; Ohba 2011; Ministry of the Environment, Government of Japan 2014; Nakajima et al. 2020). Therefore, understanding the distribution of conservation target species is essential for formulating effective conservation measures.

In recent years, environmental DNA (eDNA) analysis has emerged as a valuable tool for estimating the distribution of wildlife (Ruppert et al. 2019; Duarte et al. 2023). It has also helped monitor many aquatic insects (Doi et al. 2017; Wang et al. 2024). In addition to species-specific eDNA analysis primer design (Doi et al. 2017; Ogata et al. 2023), metabarcoding has also helped in analyzing aquatic insect fauna, contributing significantly to understanding their distribution (Takenaka et al. 2024; Wang et al. 2024).

In aquatic insects, metabarcoding using eDNA is essential for improving identification accuracy, which relies on the availability of reference sequences of each species. However, in Japan, the development of aquatic insect DNA libraries is slow (Kishimoto-Yamada et al. 2022). In the Barcode of Life Data System (BOLD), for example, of the total 13252 coleopteran and 3785 hemipteran taxa in Japan, the proportion of taxa for which sequences have been registered is 8% and 6%, respectively. Sequences of only 43 and 24 taxa were also registered for Dytiscidae and Hydrophilidae in Japan, which are the main families of aquatic beetles, respectively, in BOLD. Although the DNA barcode regions of the mitochondrial DNA (mtDNA) cytochrome *c* oxidase subunit I (COI) have been determined for several regions and taxa of aquatic insects (Inai et al. 2022; Hayashi et al. 2024a, b), they are not comprehensive enough to cover Japanese taxa. Additionally, the mtDNACOI region poses several challenges as a marker for eDNA metabarcoding analysis, including the requirement of longer sequence length (i.e., c. 650 bp) and frequent mutation of primers, which may result in non-specific amplification and amplification failure (Leese et al. 2021). Thus, there are significant issues with the marker’s data availability and accumulation for eDNA metabarcoding analysis.

Here, we focused on the mtDNA 16S rRNA region. Compared to the mtDNACOI region, it has a lower mutation rate (Marquina et al. 2019a, b; Takenaka et al. 2023) and poses a lower risk of PCR failure or pseudogene amplification in some taxonomic groups (Yano et al. 2020; Takenaka et al. 2023). Recently, a primer set targeting the mtDNA 16S rRNA region (‘MtInsects-16S’) was developed. Due to the short PCR product lengths (i.e., c. 210 bp), this primer set is considered suitable for eDNA analysis (Takenaka et al. 2023). Moreover, Takenaka et al. (2024) also elucidated its applicability for metabarcoding with eDNA in stream insects, particularly in Ephemeroptera, Plecoptera, and Trichoptera (Takenaka et al. 2024). Based on these studies, we concluded that the mtDNA 16S rRNA region is well-suited for eDNA metabarcoding analysis, and the database enrichment in aquatic coleopteran and hemipteran insects is essential.

In this study, we constructed the sequence datasets of the mtDNA 16S rRNA region for aquatic coleopteran and hemipteran insects that spend a significant portion of their life cycle in or on water. These groups are predominantly found in lentic waters in Japan and include many endangered species (Hayashi et al. 2020). The dataset is anticipated to contribute to future distribution surveys using eDNA metabarcoding and support the construction of conservation policies based on these surveys.

## ﻿Material and methods

### ﻿Target taxa and DNA sampling

This study includes the taxa listed in the ‘List of Aquatic Coleoptera and Hemiptera of Japan’ (Nakajima 2024) as the target taxa. The list comprises coleopteran 394 taxa (Chrysomelidae, Dytiscidae, Gyrinidae, Haliplidae, Hydraenidae, Hydrophiloidea, Microsporioidea, and Noteridae) and hemipteran 128 taxa (Gerromorpha and Nepomorpha), which spend most of their life cycle in water or on the surface, as documented in Japan. The scientific name of each taxon follows Nakajima (2024).

Additionally, the families Gelastocoridae and Heteroceridae were included, as listed in Nakajima et al. (2020), and the nomenclature was based on the same source (Nakajima et al. 2020). Although *Micronecta* spp. is categorized under Corixidae in Nakajima (2024), prior studies have classified it under Micronectidae, a practice we adopt here (Meyin et al. 2016; Ye et al. 2023; Xie et al. 2024).

In total, we collected 199 coleopteran taxa and 68 hemipteran taxa. As the samples collected included many endangered species, only the names of the prefectures are listed in Suppl. material 1 as collection sites. Live or freshly dead individuals were preserved in 99.5% ethanol for further analysis. The collected individuals were identified according to the following references: Kawai and Tanida (2018), Minoshima and Inahata (2019), Nakajima et al. (2020), Watanabe and Kamite (2020), Hirasawa and Yoshitomi (2021), Matsushima (2022), Karube et al. (2023), Hayashi et al. (2023), Minoshima et al. (2023), Watanabe and Hayashi (2023) and Watanabe et al. (2023). The authors double-checked the difficult-to-identify individuals.

### ﻿DNA extraction and library preparation

For individuals with a body length of approximately 5 mm or more, one middle or hind leg was cut off and used for DNA extraction, while the entire body was used for those with a body length of less than 5 mm. Genomic DNA was extracted using the Chelex method according to the protocol of Miura et al. (2017). DNA was extracted from all samples without destroying the body, and they were deposited as voucher specimens in four institutions: Aquamarine Inawashiro Kingfishers Aquarium, Hoshizaki Institute for Wildlife Protection, Ishikawa Insect Museum, and Museum of Nature and Human Activities, Hyogo (Suppl. material 1).

A single pair of primers was used to amplify the partial sequence of the mtDNA 16S rRNA region; MtInsects-16S_F: 5′-GGA CGA GAA GAC CCT WTA GA-3′ and MtInsects-16S_R: 5′-ATC CAA CAT CGA GGT CGC AA-3′ (Takenaka et al. 2023). The first PCR was performed as a 10 μl reaction volume containing 1 ng template DNA, 5 μl of 2×PCR Buffer for KOD -Multi & Epi-, 0.2 μl KOD -Multi & Epi- (TOYOBO), and 0.2 μM for each primer. The PCR cycle for the 16S rRNA region was as follows: template denaturation at 94 °C for 12 min, followed by 30 cycles of denaturation at 94 °C for 1 min, annealing at 50 °C for 1 min, and extension at 72 °C for 0.5 min; followed by a final extension at 72 °C for 3 min. Library preparation after the first PCR followed the standard MPM-seq protocol by Suyama et al. (2022). Subsequent paired-end sequencing was conducted using 2 × 250 bp cycle run on an Illumina MiSeq Sequencer (Illumina, San Diego, USA) and with the MiSeq Reagent Nano Kit v. 2 (500 cycles).

### ﻿Assembling sequences

Data pre-processing, quality control, and identification of representative sequences for the mtDNA 16S rRNA region were conducted using Claident v. 0.2.2019.05.10 (Tanabe and Toju 2013), as described by Suyama et al. (2022) and Kurata et al. (2024).

Non-demultiplexed fast files were required for quality control and data analysis using Claident. The non-demultiplexed fast files (261 bp) were generated from the BCL files using bcl2fastq v. 1.8.4 (Illumina). During this step, non-demultiplexed fast reads were sorted based on the index reads (index1: 9 bp, index2: 5 bp), and the last position of the raw reads was trimmed (--use-bases-mask Y260n,I9,I5,Y260n), as per the settings in Kurata et al. (2024). The Claident command of *clsplitseq* was then used to demultiplex the non-multiplexed fast reads, specifying the indices and primer sequences with a quality threshold of the index sequence set to 30 (--minqualtag = 30). The option of “--truncateN = enable” was included in *clsplitseq* because 0–3 Ns were added to the beginning of the primer sequences.

To identify overlaps between the forward and reverse reads, the *clconcatpair* command was used with the same settings mentioned in Kurata et al. (2024): i.e., the --mode = OVL argument was used to generate concatenated reads from the forward and reverse sequences. In addition, any low-quality reads were filtered out using the *clfilterseq* command settings from the standard MPM-seq protocol by Suyama et al. (2022): i.e., --maxplowequal = 0.1 --minqual = 27, to remove positions with a quality score lower than Q27. Finally, the *clcleanseqv* parameters were used to remove noisy and chimeric sequences with the following settings: --derepmode = FULLLENGTH --primarymaxnmismatch=0 --secondarymaxnmismatch = 1 --pnoisycluster = 0.5. The *clclasseqv* command was used to identify representative sequences for each sample and each gene, with a 99% identity threshold (--minident = 0.99). The mitochondrial 16S sequences were directly detected via the *clclasseqv* step.

### ﻿Phylogenetic analysis

The phylogenetic relationships were constructed based on the mtDNA 16S rRNA region to check the correctness of each obtained sequence of aquatic coleopteran and hemipteran insects. Mitochondrial sequence alignment was performed using MAFFT v. 7.526 L-INS-i (Katoh and Standley 2013). The maximum likelihood (ML) tree was generated using IQ-TREE v. 2.3.4 (Minh et al. 2020) and ultrafast bootstrapping (UB) with 1000 replicates. The substitution models were estimated using ModelFinder (Kalyaanamoorthy et al. 2017) within IQ-TREE v. 2.3.4 based on the BIC value. The best models chosen for each locus were GTR+F+I+G4 for coleopteran insects and TVM+F+I+G4 for hemipteran insects.

## ﻿Results and discussion

After clustering in Claident, we obtained 16–5422 reads for aquatic coleopteran (median:1,379; average:1,442) and 6–7093 reads for aquatic hemipteran insects (median, 1867; mean, 2277). The resulting phylogenetic trees were visualized and edited using FigTree v. 1.4.4. For taxa where phylogenetic analysis did not indicate monophyly (*Anacaena okinawana*, *Berosus japonicus*, *Copelatus zimmermanni*, *Elmomorphus brevicornis*, *Mesovelia miyamotoi*, *Neohydrocoptus* sp., *Peltodytes intermedius*, *Peltodytes sinensis*, and *Regimbartia attenuata*), we confirmed that all of them matched the sequences for related taxa within the same genus by BLAST searches. The number of collected reads was less than 10 for one sample of *Nerthra macrothorax* (sample ID: K043). It formed a monophyletic lineage with another sample of the same species (sample ID: K042), and the genetic differentiation was small, indicating that an accurate sequence was obtained.

**Figure 1.**
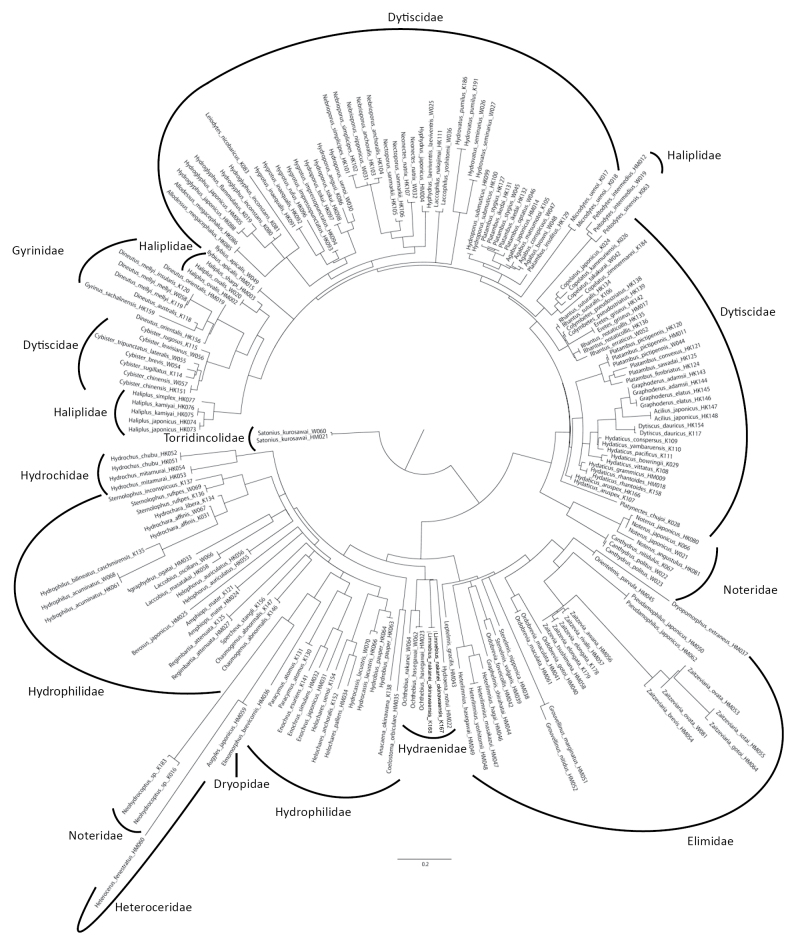
The circular phylogenetic tree of aquatic coleopteran insects in this study. Maximum likelihood (ML) phylogenetic tree based on the mtDNA 16S rRNA region.

**Figure 2.**
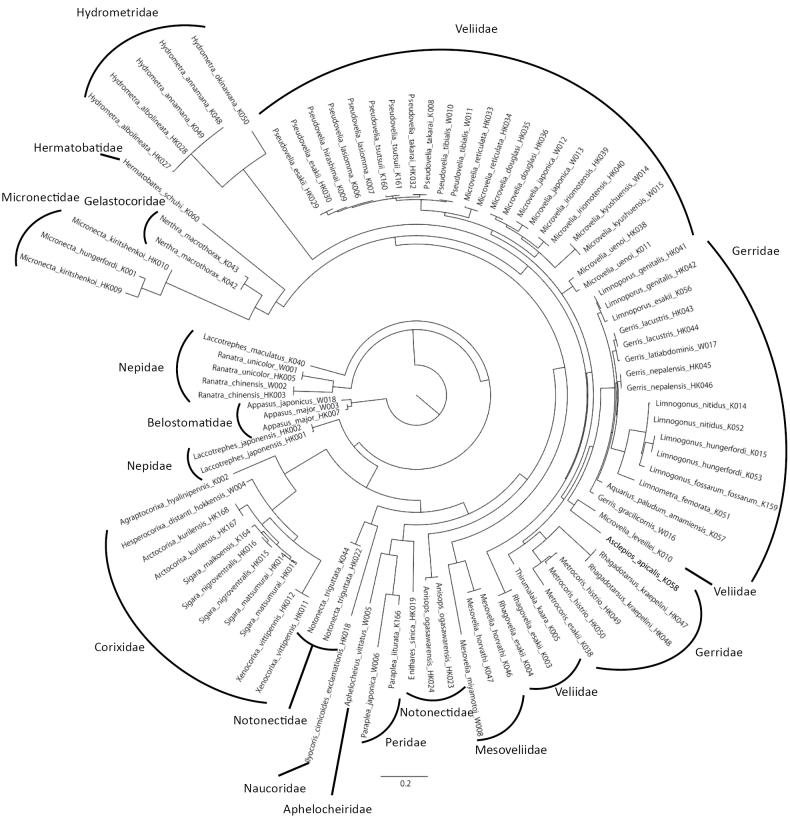
The circular phylogenetic tree of aquatic hemipteran insects in this study. Maximum likelihood (ML) phylogenetic tree based on the mtDNA 16S rRNA region.

This study successfully determined the sequences for 70.3% and 85.0% of the collected coleopteran and hemipteran taxa, respectively (Suppl. material 6). The failures in sequence identification were likely due to experimental errors or primer mismatches. Finally, we obtained 140 coleopteran taxa (including species and subspecies), representing 68 genera (Fig. 1, Suppl. materials 4, 6; Table 1, Suppl. materials 1, 2). These covered 35.5% and 72.0% of total taxa and genera of aquatic coleopteran insects in Japan. Similarly, 58 taxa were determined for aquatic Hemiptera, representing 31 genera (Fig. 2, Suppl. material 5, 6; Table 1, Suppl. material 1, 3). These accounted for 45.3% and 70.5% of total taxa and genera of aquatic hemipteran insects in Japan.

**Table 1.** Number of Japanese aquatic coleopteran and hemipteran taxa used in this study, of which sequences were obtained. The number of taxa is based on Nakajima (2024) (accessed in October 2024).

Order name	Family name	Number of taxa recorded in Japan	Number of taxa used for sequencing	Number of taxa for which sequences were obtained	Percentage of all taxa that have been sequenced
Coleoptera	Haliplidae	13	10	7	53.8%
	Noteridae	16	6	5	31.3%
	Dytiscidae	139	94	63	45.3%
	Gyrinidae	18	7	5	27.8%
	Torridincolidae	1	1	1	100.0%
	Hydraenidae	44	4	4	9.1%
	Hydrochidae	5	2	2	40.0%
	Helophoridae	5	1	1	20.0%
	Hydrophilidae (only aquatic species)	81	40	24	29.6%
	Spercheidae	1	1	1	100.0%
	Dryopidae	4	2	1	25.0%
	Elmidae	62	29	24	38.7%
	Chrysomelidae (only aquatic species)	2	0	0	0.0%
	Heteroceridae	3	2	2	66.7%
	Total	394	199	140	35.5%
Hemiptera	Nepidae	7	5	4	57.1%
	Belostomatidae	5	2	2	40.0%
	Micronectidae	9	3	2	22.2%
	Corixidae	22	8	7	31.8%
	Naucoridae	1	1	1	100.0%
	Aphelocheiridae	3	1	1	33.3%
	Notonectidae	12	3	3	25.0%
	Pleidae	3	3	2	66.7%
	Helotrephidae	1	0	0	0.0%
	Mesoveliidae	7	3	2	28.6%
	Hydrometridae	5	5	3	60.0%
	Veliidae	24	15	14	58.3%
	Gerridae	27	17	15	55.6%
	Hermatobatidae	1	1	1	100.0%
	Gelastocoridae	1	1	1	100.0%
	Total	128	68	58	45.3%
Total		522	267	198	37.9%

Although DNA barcode sequences for Japanese aquatic insects have been previously published (Inai et al. 2022; Takenaka et al. 2024), a few databases in Japan comprehensively cover coleopteran and hemipteran insects, with exceptions like Chironomidae (National Institute for Environmental Studies 2013) and Odonata insects (Kishimoto-Yamada et al. 2022). The addition of the sequences based on the mtDNA 16S rRNA region determined in this study and deposited in GenBank significantly increases the coverage of aquatic coleopteran and hemipteran insects. The expanded dataset is expected to contribute significantly to eDNA analyses in the future.

In addition to determining the mtDNA 16S rRNA region sequences of aquatic coleopteran and hemipteran insect taxa, this study prepared voucher specimens deposited in four institutions. Many undescribed coleopteran and hemipteran insects in Japan are still being discovered and described, making the phylogenetic analysis critical for taxonomic revision (Hayashi et al. 2020; Nakajima et al. 2020). On the other hand, since many of these taxa are classified as endangered (Ministry of the Environment, Government of Japan 2014), the voucher specimen collections, which include both the morphological and DNA sequence information, housed in Japanese research institutions and museums, are of utmost importance for future conservation and taxonomic efforts (Nakahama 2021).

The DNA sequences obtained in our study are intended primarily as reference sequences for DNA metabarcoding, such as eDNA analysis. These sequences, being relatively short (~210bp), are not suitable for phylogenetic analysis; therefore, they do not provide an accurate estimation of phylogenetic relationships. For example, Dytiscidae and Hydrophilidae were estimated as polyphyletic (Fig. 1, Suppl. material 4), which contradicts the phylogenetic relationships of Coleoptera estimated through genomic-level analysis (McKenna et al. 2019; Cai et al. 2022). In addition, *Heterlimnius masakazui* and *H. yoshitomii* (Elmidae) could not be distinguished as two different species using the obtained sequences. These limitations are clearly due to the short sequence length. For robust phylogenetic analysis, it is necessary to integrate these sequences with data from other loci.

In addition to eDNA analysis, the sequence data obtained would be highly valuable for other DNA metabarcoding research. For instance, it could facilitate the analysis of feeding habits from fecal samples of insectivores, ensuring molecular identification from small tissue samples (Rytkönen et al. 2019; Ingala et al. 2021). Our comprehensive DNA sequence dataset would enhance the detailed identification of food organism taxa. Furthermore, it could aid in the molecular identification of samples with degraded and fragmented DNA, such as museum specimens or parts of a deceased organism found in the wild. This is because PCR is more likely to succeed when primers targeting short PCR products are used. For example, Nakahama and Isagi (2017) showed that PCR was successfully performed on more than 80% of 30-year-old insect specimens using primers with c. 160 bp PCR product length. The PCR product length of the primer pair (MtInsects-16S_F and MtInsects-16S_R) used in this study is c. 210 bp, which is slightly longer than the PCR product length of the primers used in Nakahama and Isagi (2017). However, it is likely to be much more successful for PCR of DNA in old specimens and feces compared to primers in the COI region, where the PCR product length exceeds 650 bp. Our sequence dataset is expected to be applicable not only for eDNA analysis but also for a variety of molecular research studies.

There are two main limitations for the future DNA sequence dataset of Japanese aquatic coleopteran and hemipteran insects. The first limitation is the need for comprehensive enrichment of the sequence dataset. This study provided the data for just under 40% of Japanese aquatic coleopteran and hemipteran insects. However, a significant number of endangered species, such as *Kirkaldyia deyrolli* (Belostomatidae) and *Dytiscus sharpi* (Dytiscidae) (Ministry of the Environment, Government of Japan 2014), remain unsequenced. Further studies are essential to enrich the dataset. The second limitation concerns the determination of longer sequences of the mtDNA 16S rRNA region. Although mtDNA 16S rRNA region primers targeting a longer PCR product were previously designed (AQdb-16S_F and AQdb-16S_R) (Takenaka et al. 2023), this study used primers targeting a shorter PCR product (MtInsects-16S_F and MtInsects-16S_R) to accommodate the MiSeq Reagent Nano Kit v.2 (500 cycles) used for sequencing. Recent advancements in high-throughput sequencing technologies, such as Nanopore, which allow the determination of longer sequences, have facilitated the construction of various DNA barcoding databases (Menegon et al. 2017; Hebert et al. 2023). Nanopore sequencing has lower sequencing accuracy than other sequencers, although accuracy has been improving rapidly over the last several years (Kerkhof 2021). Moving forward, the construction of the dataset by determining longer DNA sequences would enhance the utility, not only for eDNA analysis but also for phylogenetic analysis.

The database of mtDNA 16S rRNA region sequences of aquatic coleopteran and hemipteran insect taxa obtained in this study would contribute to metabarcoding, including eDNA analysis. These aquatic insects include many endangered species, including many small species less than 1 cm in body length (Ministry of the Environment, Government of Japan 2014). Many of these species are difficult for beginners to identify (Nakajima et al. 2020). Even for larger species, comprehensive distribution surveys in many habitats across large areas could be time-consuming. Metabarcoding of environmental DNA would facilitate the detection of diverse taxa, including small to large species. It would also contribute to the understanding of food webs in wetland ecosystems through diet analysis by metabarcoding (Huang et al. 2022). The present study covered more than 70% of the Japanese genera in aquatic coleopteran and hemipteran insects. The use of this database for future studies using metabarcoding in wetland ecosystems is expected to greatly advance the identification of species and genera of sequences previously considered unknown taxa.

## ﻿Additional information

### Use of AI

No use of AI was reported.

### Author ORCIDs

Naoyuki Nakahama https://orcid.org/0000-0003-3106-8289

Kei Hirasawa https://orcid.org/0009-0008-2296-3699

Masaya Kato https://orcid.org/0009-0008-3878-4898

Kohei Watanabe https://orcid.org/0000-0002-8761-232X

Seikan Kurata https://orcid.org/0000-0003-3825-3938

Masakazu Hayashi https://orcid.org/0000-0003-3708-9005

### Data availability

All of the data that support the findings of this study are available in the main text or Supplementary Information.

## Supplementary materials

Supplementary material 1 Sample list used in this study (including both scientific and Japanese names) Data type xlsx Explanation note In each sample, the collection site and date, and collector are indicated. For the collection sites, only the prefecture is listed for conservation perspectives. The number of reads obtained is described for taxa for which phylogenetic analyses were conducted and registered in GenBank. https://binary.pensoft.net/file/1422297This dataset is made available under the Open Database License (http://opendatacommons.org/licenses/odbl/1.0/). The Open Database License (ODbL) is a license agreement intended to allow users to freely share, modify, and use this Dataset while maintaining this same freedom for others, provided that the original source and author(s) are credited.Naoyuki Nakahama, Kei Hirasawa, Masaya Kato, Kohei Watanabe, Seikan Kurata, Masakazu Hayashi

Supplementary material 2 A list of each of the aquatic coleopteran insects treated in this study for each family (including both scientific and Japanese names) Data type xlsx Explanation note Sample IDs are given for taxa for which sequences were obtained. https://binary.pensoft.net/file/1422298This dataset is made available under the Open Database License (http://opendatacommons.org/licenses/odbl/1.0/). The Open Database License (ODbL) is a license agreement intended to allow users to freely share, modify, and use this Dataset while maintaining this same freedom for others, provided that the original source and author(s) are credited.Naoyuki Nakahama, Kei Hirasawa, Masaya Kato, Kohei Watanabe, Seikan Kurata, Masakazu Hayashi

Supplementary material 3 A list of each of the aquatic hemipteran insects treated in this study, for each family (including both scientific and Japanese names) Data type xlsx Explanation note Sample IDs are given for taxa for which sequences were obtained. https://binary.pensoft.net/file/1422299This dataset is made available under the Open Database License (http://opendatacommons.org/licenses/odbl/1.0/). The Open Database License (ODbL) is a license agreement intended to allow users to freely share, modify, and use this Dataset while maintaining this same freedom for others, provided that the original source and author(s) are credited.Naoyuki Nakahama, Kei Hirasawa, Masaya Kato, Kohei Watanabe, Seikan Kurata, Masakazu Hayashi

Supplementary material 4 The phylogenetic tree of aquatic coleopteran insects in this study Data type pdf Explanation note Maximum likelihood (ML) phylogenetic tree based on the mtDNA 16S rRNA region. The node numbers represent ML ultrabootstrap values. In each branch, only values with ultrabootstrap > 70 are denoted in the tree. https://binary.pensoft.net/file/1422300This dataset is made available under the Open Database License (http://opendatacommons.org/licenses/odbl/1.0/). The Open Database License (ODbL) is a license agreement intended to allow users to freely share, modify, and use this Dataset while maintaining this same freedom for others, provided that the original source and author(s) are credited.Naoyuki Nakahama, Kei Hirasawa, Masaya Kato, Kohei Watanabe, Seikan Kurata, Masakazu Hayashi

Supplementary material 5 The phylogenetic tree of aquatic hemipteran insects in this study Data type pdf Explanation note Maximum likelihood (ML) phylogenetic tree based on the mtDNA 16S rRNA region. The node numbers represent ML ultrabootstrap values. In each branch, only values with ultrabootstrap > 70 are denoted in the tree. https://binary.pensoft.net/file/1422301This dataset is made available under the Open Database License (http://opendatacommons.org/licenses/odbl/1.0/). The Open Database License (ODbL) is a license agreement intended to allow users to freely share, modify, and use this Dataset while maintaining this same freedom for others, provided that the original source and author(s) are credited.Naoyuki Nakahama, Kei Hirasawa, Masaya Kato, Kohei Watanabe, Seikan Kurata, Masakazu Hayashi

Supplementary material 6 The fasta format file containing the sequences of mtDNA 16S rRNA region of all aquatic coleopteran and hemipteran insects determined in this study. Data type fasta Explanation note In each sequence, the scientific name and sample ID are described. https://binary.pensoft.net/file/1422302This dataset is made available under the Open Database License (http://opendatacommons.org/licenses/odbl/1.0/). The Open Database License (ODbL) is a license agreement intended to allow users to freely share, modify, and use this Dataset while maintaining this same freedom for others, provided that the original source and author(s) are credited.Naoyuki Nakahama, Kei Hirasawa, Masaya Kato, Kohei Watanabe, Seikan Kurata, Masakazu Hayashi
