# Supplementary figures and images for: ﻿Mitochondrial DNA 16S region and voucher specimen collection of Japanese aquatic Coleoptera and Hemiptera for environmental DNA metabarcoding analyses
Source: Zookeys. 2025 Sep 23;1253:103–19. doi: 10.3897/zookeys.1253.146226 (PMC12485484; doi:10.3897/zookeys.1253.146226)

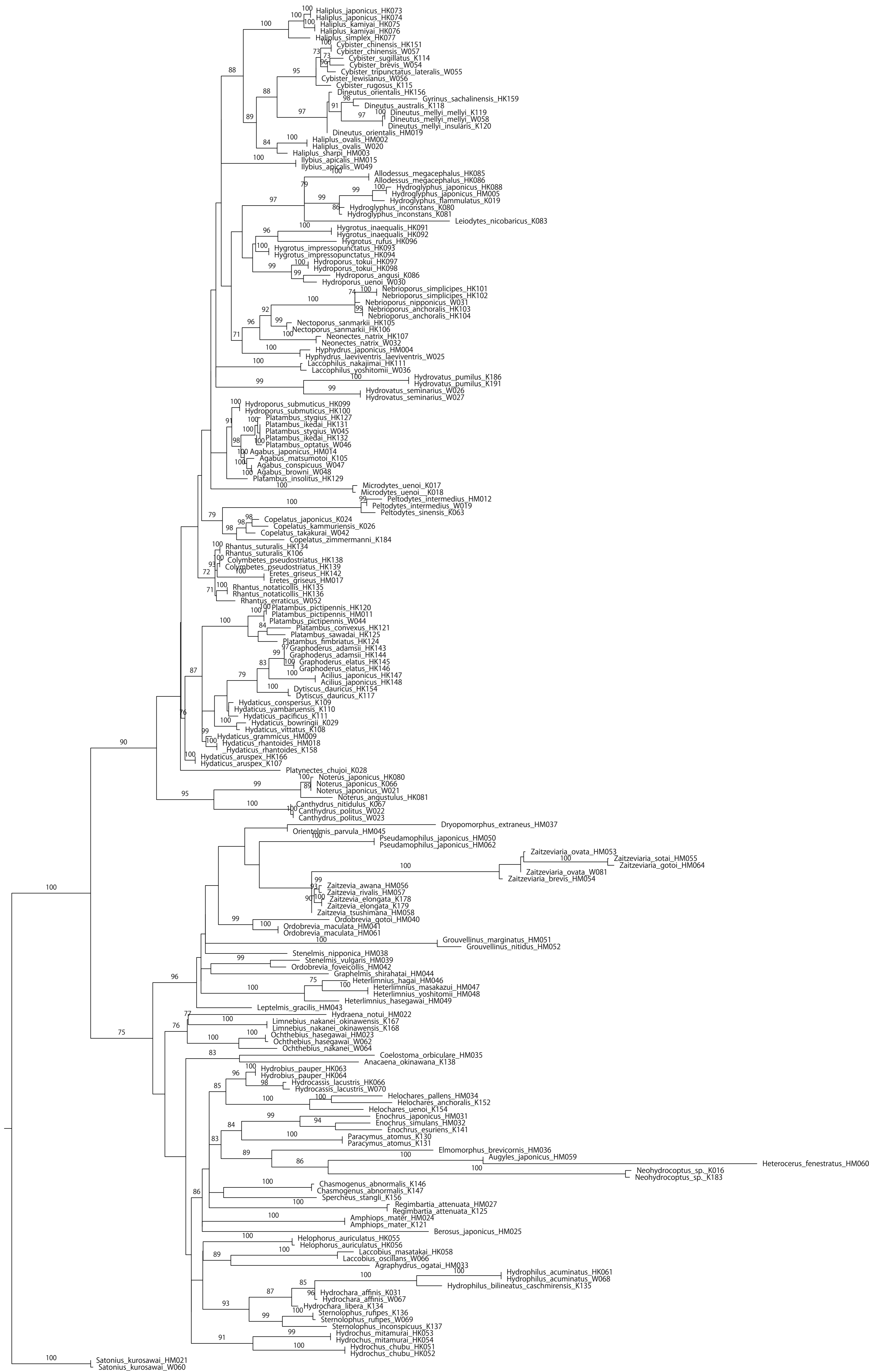

Supplement: Supplementary material 4 — The phylogenetic tree of aquatic coleopteran insects in this study [file zookeys-1253-103_article-146226__-s004.pdf]

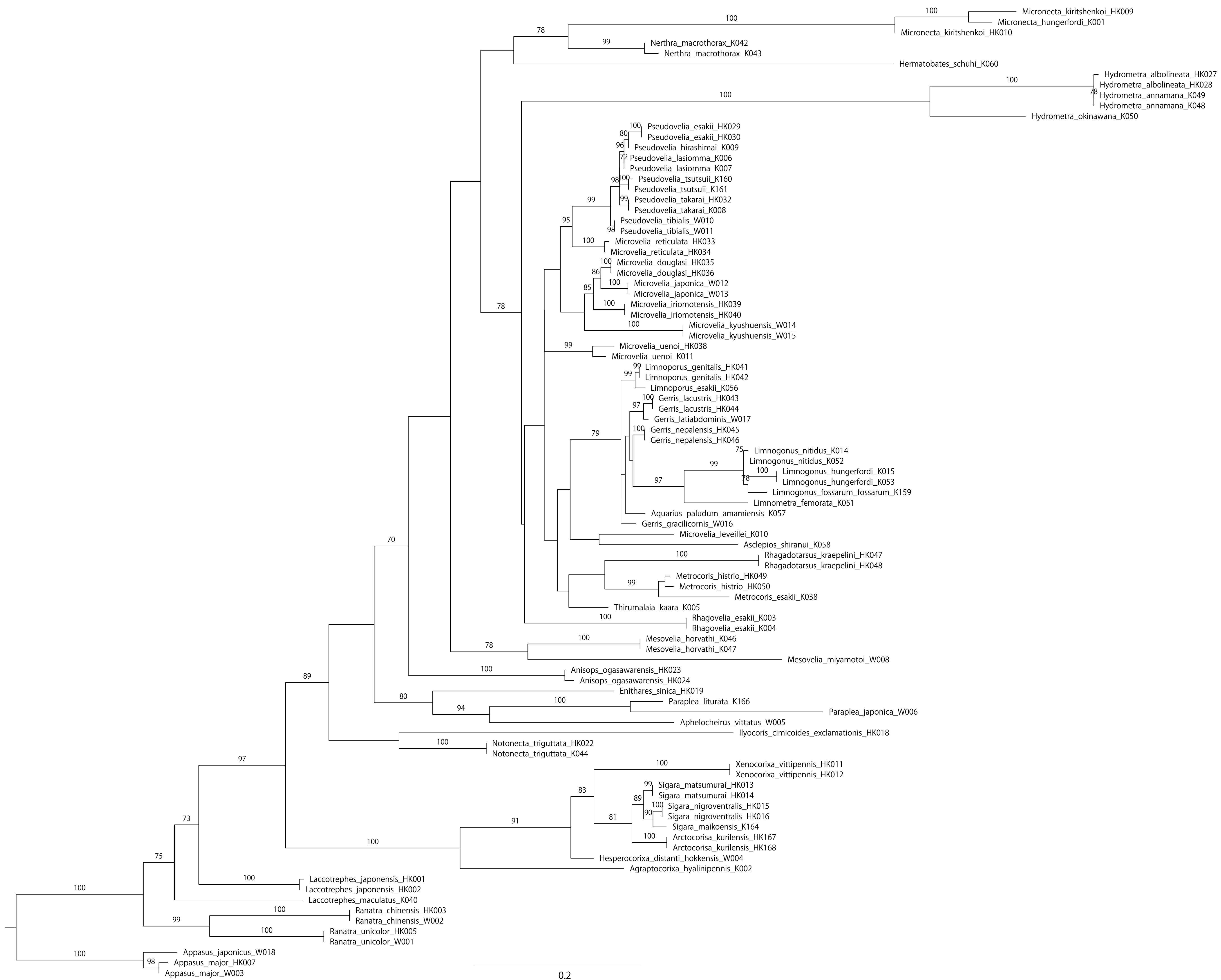

Supplement: Supplementary material 5 — The phylogenetic tree of aquatic hemipteran insects in this study [file zookeys-1253-103_article-146226__-s005.pdf]
